# Severe Acute Respiratory Syndrome Coronavirus 2 from Patient with Coronavirus Disease, United States

**DOI:** 10.3201/eid2606.200516

**Published:** 2020-06

**Authors:** Jennifer Harcourt, Azaibi Tamin, Xiaoyan Lu, Shifaq Kamili, Senthil K. Sakthivel, Janna Murray, Krista Queen, Ying Tao, Clinton R. Paden, Jing Zhang, Yan Li, Anna Uehara, Haibin Wang, Cynthia Goldsmith, Hannah A. Bullock, Lijuan Wang, Brett Whitaker, Brian Lynch, Rashi Gautam, Craig Schindewolf, Kumari G. Lokugamage, Dionna Scharton, Jessica A. Plante, Divya Mirchandani, Steven G. Widen, Krishna Narayanan, Shinji Makino, Thomas G. Ksiazek, Kenneth S. Plante, Scott C. Weaver, Stephen Lindstrom, Suxiang Tong, Vineet D. Menachery, Natalie J. Thornburg

**Affiliations:** Centers for Disease Control and Prevention, Atlanta, Georgia, USA (J. Harcourt, A. Tamin, X. Lu, K. Queen, Y. Tao, C.R. Paden, Y. Li, C. Goldsmith, B. Whitaker, R. Gautam, S. Lindstrom, S. Tong, N.J. Thornburg);; Eagle Medical Services, Atlanta (S. Kamili, S.K. Sakthivel, J. Murray, B. Lynch);; IHRC, Atlanta (J. Zhang, H. Wang); Oak Ridge Institute for Science and Education, Oak Ridge, Tennessee, USA (A. Uehara);; Synergy America, Inc., Atlanta (H.A. Bullock, L. Wang);; University of Texas Medical Branch, Galveston, Texas, USA (C. Schindewolf, K.G. Lokugamage, D. Mirchandani, S. Widen, K. Narayanan, S. Makino, T.G. Ksiazek, S.C. Weaver, V.D. Menachery);; World Reference Center for Emerging Viruses and Arboviruses, Galveston (D. Scharton, J.A. Plante, T.G. Ksiazek, K.S. Plante, S.C. Weaver, V.D. Menachery)

**Keywords:** coronavirus, viruses, severe acute respiratory syndrome coronavirus 2, SARS-CoV-2, 2019 novel coronavirus disease, COVID-19, respiratory infections, PCR, zoonoses, isolation, characterization, United States

## Abstract

The etiologic agent of an outbreak of pneumonia in Wuhan, China, was identified as severe acute respiratory syndrome coronavirus 2 in January 2020. A patient in the United States was given a diagnosis of infection with this virus by the state of Washington and the US Centers for Disease Control and Prevention on January 20, 2020. We isolated virus from nasopharyngeal and oropharyngeal specimens from this patient and characterized the viral sequence, replication properties, and cell culture tropism. We found that the virus replicates to high titer in Vero-CCL81 cells and Vero E6 cells in the absence of trypsin. We also deposited the virus into 2 virus repositories, making it broadly available to the public health and research communities. We hope that open access to this reagent will expedite development of medical countermeasures.

A novel coronavirus, severe acute respiratory syndrome coronavirus 2 (SARS-CoV-2), has been identified as the source of a pneumonia outbreak in Wuhan, China, in late 2019 (*1*,*2*). The virus was found to be a member of the β coronavirus family, in the same species as SARS-CoV and SARS-related bat CoVs (*3*,*4*). Patterns of spread indicate that SARS-CoV-2 can be transmitted person-to-person, and may be more transmissible than SARS-CoV (*5*–*7*). The spike protein of coronaviruses mediates virus binding and cell entry. Initial characterization of SARS-CoV-2 spike indicates that it binds the same receptor as SARS-CoV angiotensin-converting enzyme, which is expressed in both upper and lower human respiratory tracts (*8*).

The unprecedented rapidity of spread of this outbreak represents a critical need for reference reagents. The public health community requires viral lysates to serve as diagnostic references, and the research community needs virus isolates to test antiviral compounds, develop new vaccines, and perform basic research. In this article, we describe isolation of SARS-CoV-2 from a patient who had coronavirus disease (COVID-19) in the United States and described its genomic sequence and replication characteristics. We have made the virus isolate available to the public health community by depositing it into 2 virus reagent repositories.

## Methods

### Specimen Collection

Virus isolation from patient samples was deemed not to be human subjects research by the National Center for Immunizations and Respiratory Diseases, Centers for Disease Control and Prevention (CDC) (research determination no. 0900f3eb81ab4b6e). Clinical specimens from a case-patient who had acquired COVID-19 during travel to China and who was identified in Washington, USA, were collected as described (*1*). Nasopharyngeal (NP) and oropharyngeal (OP) swab specimens were collected on day 3 postsymptom onset, placed in 2–3 mL of viral transport medium, used for molecular diagnosis, and frozen. Confirmed PCR-positive specimens were aliquoted and refrozen until virus isolation was initiated.

### Cell Culture, Limiting Dilution, and Virus Isolation

We used Vero CCL-81 cells for isolation and initial passage. We cultured Vero E6, Vero CCL-81, HUH 7.0, 293T, A549, and EFKB3 cells in Dulbecco minimal essential medium (DMEM) supplemented with heat-inactivated fetal bovine serum (5% or 10%) and antibiotics/antimycotics (GIBCO, https://www.thermofisher.com). We used both NP and OP swab specimens for virus isolation. For isolation, limiting dilution, and passage 1 of the virus, we pipetted 50 μL of serum-free DMEM into columns 2–12 of a 96-well tissue culture plate, then pipetted 100 μL of clinical specimens into column 1 and serially diluted 2-fold across the plate. We then trypsinized and resuspended Vero cells in DMEM containing 10% fetal bovine serum, 2× penicillin/streptomycin, 2× antibiotics/antimycotics, and 2× amphotericin B at a concentration of 2.5 × 10^5^ cells/mL. We added 100 μL of cell suspension directly to the clinical specimen dilutions and mixed gently by pipetting. We then grew the inoculated cultures in a humidified 37°C incubator in an atmosphere of 5% CO_2_ and observed for cytopathic effects (CPEs) daily. We used standard plaque assays for SARS-CoV-2, which were based on SARS-CoV and Middle East respiratory syndrome coronavirus (MERS-CoV) protocols (*9*,*10*).

When CPEs were observed, we scraped cell monolayers with the back of a pipette tip. We used 50 μL of viral lysate for total nucleic acid extraction for confirmatory testing and sequencing. We also used 50 μL of virus lysate to inoculate a well of a 90% confluent 24-well plate.

### Inclusivity/Exclusivity Testing

From the wells in which CPEs were observed, we performed confirmatory testing by using real-time reverse transcription PCR (CDC) and full-genome sequencing (*1*). The CDC molecular diagnostic assay targets 3 portions of the nucleocapsid gene, and results for all 3 portions must be positive for a sample to be considered positive (https://www.cdc.gov/coronavirus/2019-ncov/lab/rt-pcr-detection-instructions.html and https://www.cdc.gov/coronavirus/2019-ncov/lab/rt-pcr-panel-primer-probes.html). To confirm that no other respiratory viruses were present, we performed Fast Track Respiratory Pathogens 33 Testing (FTD Diagnostics, http://www.fast-trackdiagnostics.com).

### Whole-Genome Sequencing

We designed 37 pairs of nested PCRs spanning the genome on the basis of the coronavirus reference sequence (GenBank accession no. NC045512). We extracted nucleic acid from isolates and amplified by using the 37 individual nested PCRs. We used positive PCR amplicons individually for subsequent Sanger sequencing and also pooled them for library preparation by using a ligation sequencing kit (Oxford Nanopore Technologies, https://nanoporetech.com), subsequently for Oxford Nanopore MinION sequencing. We generated consensus nanopore sequences by using Minimap version 2.17 (https://github.com) and Samtools version 1.9 (http://www.htslib.org). We generated consensus sequences by Sanger sequencing from both directions by using Sequencher version 5.4.6 (https://www.genecodes.com), and further confirmed them by using consensus sequences generated from nanopore sequencing.

To sequence passage 4 stock, we prepared libraries for sequencing by using the Next Ultra II RNA Prep Kit (New England Biolabs, https://www.neb.com) according to the manufacturer’s protocol. In brief, we fragmented ≈70–100 ng of RNA for 15 min, followed by cDNA synthesis, end repair, and adaptor ligation. After 6 rounds of PCR, we analyzed libraries by using an Agilent Bioanalyzer (https://www.agilent.com) and quantified them by using a quantitative PCR. We pooled samples and sequenced samples by using a paired-end 75-base protocol on an Illumina (Illumina, Inc., https://www.illumina.com) MiniSeq instrument and using the High-Output Kit and then processed reads by using Trimmomatic version 0.36 (*11*) to remove low-quality base calls and any adaptor sequences. We used the de novo assembly program ABySS (*12*) to assemble the reads into contigs by using several different sets of reads and kmer values ranging from 20 to 40. We compared contigs >400 bases against the National Center for Biotechnology Information (Bethesda, MD, USA) nucleotide collection using BLAST (https://blast.ncbi.nlm.nih.gov). A nearly full-length viral contig obtained in each sample had 100% identity to the 2019-nCoV/USA-WA1/2020 strain (GenBank accession no. MN985325.1). All the remaining contigs mapped to either host cell rRNA or mitochondria. We mapped the trimmed reads to the reference sequence by using BWA version 0.7.17 (*13*) and visualized these reads by using the Integrated Genomics Viewer (*14*) to confirm the identity with the USA-WA1/2020 strain.

### Electron Microscopy

We scraped infected Vero cells from the flask, pelleted by low-speed centrifugation, rinsed with 0.1 mol/L phosphate buffer, pelleted again, and fixed for 2 h in 2.5% buffered glutaraldehyde. We then postfixed specimens with 1% osmium tetroxide, en bloc stained with 4% uranyl acetate, dehydrated, and embedded in epoxy resin. We cut ultrathin sections, stained them with 4% uranyl acetate and lead citrate, and examined them by using a Thermo Fisher/FEI Tecnai Spirit electron microscope (https://www.fei.com).

### Protein Analysis and Western Blotting

We harvested cell lysates by using Laemmli sodium dodecyl sulfate–polyacrylamide gel electrophoresis sample buffer (Bio-Rad, https://www.bio-rad.com) containing 2% SDS and 5% β-mercaptoethanol. We removed the cell lysates from a Biosafety Level 3 Laboratory, boiled them, and load them onto a polyacrylamide gel. We subjected the lysates to sodium dodecyl sulfate–polyacrylamide gel electrophoresis, followed by transfer to a polyvinylidene difluoride polyvinylidene fluoride membrane. We then blocked the membrane in 5% nonfat dry milk dissolved in Tris-buffered saline containing 0.1% Tween-20 (TBS-T) for 1 h, followed by a short wash with TBS-T. We incubated the membrane overnight with primary antibody, either rabbit polyclonal serum against the SARS-CoV spike protein (#40150-T52; Sino Biological, https://www.sinobiological.com), β-actin antibody (#4970; Cell Signaling Technology, https://www.cellsignal.com), or a custom rabbit polyclonal serum against SARS-CoV nucleocapsid. We then washed the membrane with 3 times with TBS-T and applied horseradish peroxidase-conjugated secondary antibody for 1 h. Subsequently, we washed the membrane 3 times with TBS-T, incubated with Clarity Western ECL Substrate (#1705060S; Bio-Rad), and imaged with a multipurpose imaging system.

### Generation of SARS-CoV Nucleocapsid Antibodies

We used the plasmid pBM302 (*15*) to express SARS-CoV nucleocapsid protein, with a C-terminal His6 tag, to high levels within the inclusion bodies of *Escherichia coli* and the recombinant protein was purified from the inclusion bodies by using nickel-affinity column chromatography under denaturing conditions. We used stepwise dialysis against Tris/phosphate buffer to refold the recombinant SARS-CoV nucleocapsid protein with decreasing concentrations of urea to renature the protein. We then immunized rabbits with the renatured, full-length, SARS-CoV nucleocapsid protein to generate an affinity-purified rabbit anti–SARS-CoV nucleocapsid protein polyclonal antibody.

## Results

**Figure 1:**
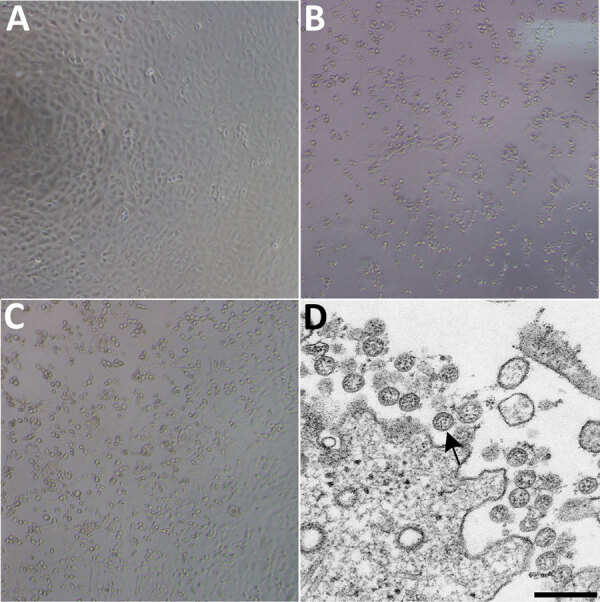
Cytopathic effect caused by severe acute respiratory syndrome coronavirus 2 from patient with coronavirus disease, United States, 2020. A–C) Phase-contrast microscopy of Vero cell monolayers at 3 days postinoculation: A) Mock, B) nasopharyngeal specimen, C) oropharyngeal specimen. Original magnifications ×10). D) Electron microscopy of virus isolate showing extracellular spherical particles with cross-sections through the nucleocapsids (black dots). Arrow indicates a coronavirus virion budding from a cell. Scale bar indicates 200 nm.

A patient was identified with confirmed COVID-19 in Washington State on January 22, 2020. CPE was not observed in mock infected cells (Figure 1, panel A). Cycle threshold (C_t_) values were 18–20 for NP specimens and 21–22 for OP specimens (*1*). The positive clinical specimens were aliquoted and refrozen inoculated into cell culture on January 22, 2020. We observed CPE 2 days postinoculation and harvested viral lysate on day 3 postinoculation (Figure 1, panels B, C). We used 50 μL of passage 1 viral lysates for nucleic acid extraction to confirm the presence of SARS-CoV-2 by using the CDC molecular diagnostic assay (*1*). The C_t_ values of 3 nucleic acid extractions were 16.0–17.1 for nucleocapsid portion 1, 15.9–17.1 for nucleocapsid portion 2, and 16.2–17.3 for nucleocapsid portion 3, which confirmed isolation of SARS-CoV-2 (C_t_ <40 is considered a positive result). We also tested extracts for 33 additional different respiratory pathogens by using the Fast Track 33 Assay. No other pathogens were detected. Identity was additionally supported by thin-section electron microscopy (Figure 1, panel D). We observed a morphology and morphogenesis characteristic of coronaviruses.

We used isolates from the first passage of an OP and an NP specimen for whole-genome sequencing. The genomes from the NP specimen (GenBank accession MT020880) and OP specimen (GenBank accession no. MT020881) showed 100% identity with each other. The isolates also showed 100% identity with the corresponding clinical specimen (GenBank accession no. MN985325).

After the second passage, we did not culture OP and NP specimens separately. We passaged virus isolate 2 more times in Vero CCL-81 cells and titrated by determining the 50% tissue culture infectious dose (TCID_50_). Titers were 8.65 × 10^6^ TCID_50_/mL for the third passage and 7.65 × 10^6^ TCID_50_/mL for the fourth passage.

We passaged this virus in the absence of trypsin. The spike protein sequence of SARS-CoV-2 has an RRAR insertion at the S1-S2 interface that might be cleaved by furin (*16*). Highly pathogenic avian influenza viruses have highly basic furin cleavage sites at the hemagglutinin protein HA1-HA2 interface that permit intracellular maturation of virions and more efficient viral replication (*17*). The RRAR insertion in SARS-CoV-2 might serve a similar function.

**Figure 2:**
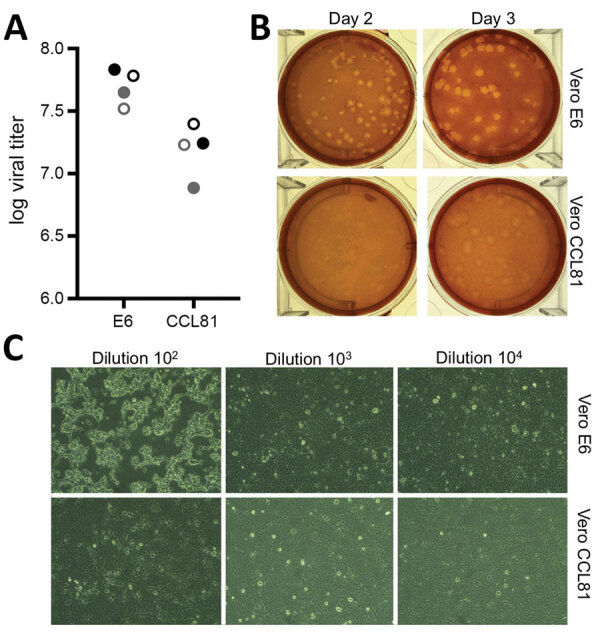
Viral propagation and quantitation of severe acute respiratory syndrome coronavirus 2 from patient with coronavirus disease, United States, 2020. A) Two virus passage 4 stocks (black and gray circles) were quantified by using plaque assay at day 2 (solid circles) and day 3 (open circles) postinfection of Vero E6 and Vero CCL81 cells. B) Plaque morphology for virus on Vero E6 and Vero CCL81 at day 2 and day 3 postinoculation. C) Cell monolayers 2 days postinfection of Vero E6 (top) and Vero CCL81 (bottom) at 3 dilutions. Original magnifications ×40.

We subsequently generated a fourth passage stock of SARS-CoV-2 on VeroE6 cells, another fetal rhesus monkey kidney cell line. We sequenced viral RNA from SARS-CoV-2 passage 4 stock and confirmed it to have no nucleotide mutations compared with the original reference sequence (GenBank accession no. MN985325). SARS-CoV has been found to grow well on VeroE6 cells and MERS-CoV on Vero CCL81 cells (*18**,**19*). To establish a plaque assay and determine the preferred Vero cell type for quantification, we titered our passage 4 stock on VeroE6 and VeroCCL81 cells. After infection with a dilution series, SARS-CoV-2 replicated in both Vero cell types; however, the viral titers were slightly higher in VeroE6 cells than in Vero CCL81 cells (Figure 2, panel A). In addition, plaques were more distinct and visible on Vero E6 cells (Figure 2, panel B). As early as 2 days postinoculation, VeroE6 cells produced distinct plaques visible by staining with neutral red. In contrast, Vero CCL81 cells produced less clear plaques and was most easily quantitated by staining with neutral red 3 days postinoculation. On the individual plaque monolayers, SARS-CoV-2 infection of Vero E6 cells produced CPE with areas of cell clearance (Figure 2, panel C). In contrast, Vero CCL81 cells had areas of dead cells that had fused to form plaques, but the cells did not clear. Together, these results suggest that VeroE6 cells might be the best choice for amplification and quantification, but both Vero cell types support amplification and replication of SARS-CoV-2.

**Figure 3:**
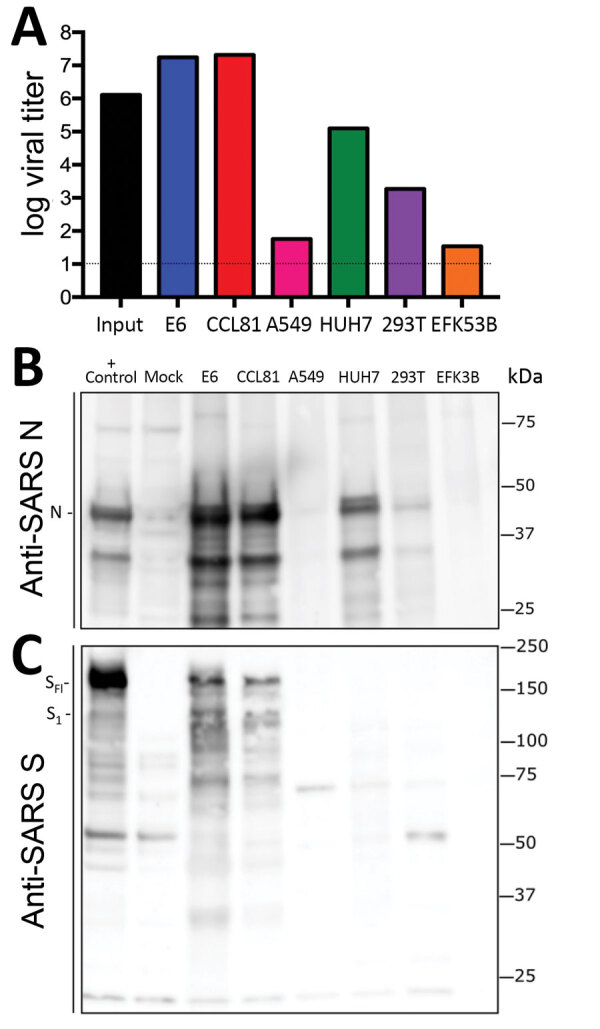
Cell lines from patient with coronavirus disease, United States, 2020, susceptible to SARS coronavirus 2 (SARS-CoV-2). Cell lines were infected with a high multiplicity of infection (>5), washed after adsorption, and subsequently harvested 24 h postinfection for viral titer and protein lysates. A) Viral titer for SARS-CoV-2 quantitated by plaque assay on Vero E6 cells 2 days postinoculation. Infected cell protein lysates were probed by using Western blotting with B) rabbit polyclonal anti-SARS N antibody or C) anti–SARS-CoV S protein antibody. Full-length spike protein (S_FL_) and spike protein S1 (S_1_) are indicated. N, nucleocapsid; S, spike protein; SARS, severe acute respiratory syndrome.

Because research has been initiated to study and respond to SARS-CoV-2, information about cell lines and types susceptible to infection is needed. Therefore, we examined the capacity of SARS-CoV-2 to infect and replicate in several common primate and human cell lines, including human adenocarcinoma cells (A549), human liver cells (HUH7.0), and human embryonic kidney cells (HEK-293T), in addition to Vero E6 and Vero CCL81 cells. We also examined an available big brown bat kidney cell line (EFK3B) for SARS-CoV-2 replication capacity. Each cell line was inoculated at high multiplicity of infection and examined 24 h postinfection (Figure 3, panel A). No CPE was observed in any of the cell lines except in Vero cells, which grew to >10^7^ PFU at 24 h postinfection. In contrast, HUH7.0 and 293T cells showed only modest viral replication, and A549 cells were incompatible with SARS-CoV-2 infection. These results are consistent with previous susceptibility findings for SARS-CoV and suggest other common culture systems, including MDCK, HeLa, HEP-2, MRC-5 cells, and embryonated eggs, are unlikely to support SARS-CoV-2 replication (*20*–*22*). In addition, SARS-CoV-2 did not replicate in bat EFK3B cells, which are susceptible to MERS-CoV. Together, the results indicate that SARS-CoV-2 maintains a similar profile to SARS-CoV in terms of susceptible cell lines.

Having established robust infection with SARS-CoV-2 in several cell types, we next evaluated the cross-reactivity of SARS-CoV antibodies against the SARS-CoV-2. Cell lysates from infected cell lines were probed for protein analysis; we found that polyclonal serum against the SARS-CoV spike protein and nucleocapsid proteins recognize SARS-CoV-2 (Figure 3, panels B, C). The nucleocapsid protein, which is highly conserved across the group 2B family, retains >90% amino acid identity between SARS-CoV and SARS-CoV-2. Consistent with the replication results (Figure 3, panel A), SARS-CoV-2 showed robust nucleocapsid protein in both Vero cell types, less protein in HUH7.0 and 293T cells, and minimal protein in A549 and EFK3B cells (Figure 3, panel B). The SARS-CoV spike protein antibody also recognized SARS-CoV-2 spike protein, indicating cross-reactivity (Figure 3, panel C). Consistent with SARS CoV, several cleaved and uncleaved forms of the SARS-CoV-2 spike protein were observed. The cleavage pattern of the SARS spike positive control from Calu3 cells, a respiratory cell line, varies slightly and could indicate differences between proteolytic cleavage of the spike proteins between the 2 viruses because of a predicted insertion of a furin cleavage site in SARS-CoV-2 (*16*). However, differences in cell type and conditions complicate this interpretation and indicate the need for further study in equivalent systems. Overall, the protein expression data from SARS-CoV nucleocapsid and spike protein antibodies recapitulate replication findings and indicate that SARS-CoV reagents can be used to characterize SARS-CoV-2 infection.

**Figure 4:**
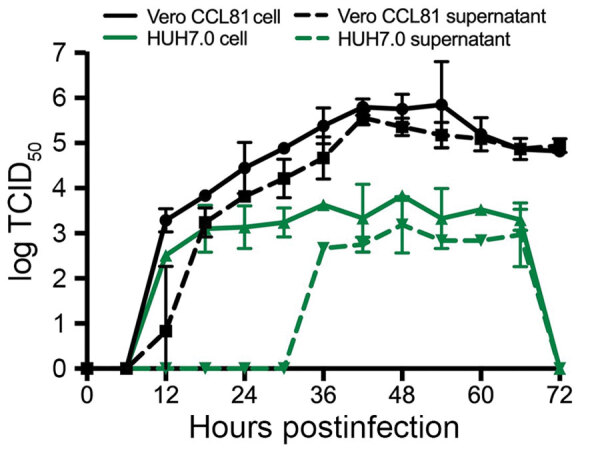
Multistep growth curve for severe acute respiratory syndrome coronavirus 2 from patient with coronavirus disease, United States, 2020. Vero CCL81 (black) and HUH7.0 cells (green) were infected at a multiplicity of infection of 0.1, and cells (solid line) and supernatants (dashed line) were harvested and assayed for viral replication by using TCID_50._ Circles, Vero CCL81 cells; squares, Vero CCL81 supernatants; triangles, HUH7.0 cells; inverted triangles, HUH7.0 supernatants. Error bars indicate SEM. TCID_50_, 50% tissue culture infectious dose.

Finally, we evaluated the replication kinetics of SARS-CoV-2 in a multistep growth curve. In brief, we infected Vero CCL-81 and HUH7.0 cells with SARS-CoV-2 at a low multiplicity of infection (0.1) and evaluated viral replication every 6 h for 72 h postinoculation, with separate harvests in the cell-associated and supernatant compartments (Figure 4). Similar to SARS-CoV, SARS-CoV-2 replicated rapidly in Vero cells after an initial eclipse phase, achieving 10^5^ TCID_50_/mL by 24 h postinfection and peaking at >10^6^ TCID_50_/mL. We observed similar titers in cell-associated and supernatant compartments, which indicated efficient egress. Despite peak viral titers by 48 h postinoculation, major CPE was not observed until 60 h postinoculation and peaked at 72 h postinoculation, indicating that infected monolayers should be harvested before peak CPE is observed. Replication in HUH7.0 cells also increased quickly after an initial eclipse phase but plateaued by 24 h postinoculation in the intracellular compartment at 2 × 10^3^ TCID_50_/mL and decreased after 66 h postinoculation. Virus was not detected in the supernatant of infected HUH7 cells until 36 h postinoculation and exhibited lower titers at all timepoints (Figure 4). Major CPE was never observed in HUH7.0 cells. These results are consistent with previous reports for SARS-CoV and MERS-CoV, which suggested similar replication dynamics between the zoonotic CoV strains (*23**,**24*).

## Discussion

We have deposited information on the SARS-CoV-2 USA-WA1/2020 viral strain described here into the Biodefense and Emerging Infections Research Resources Repository (https://www.beiresources.org) reagent resources (American Type Culture Collection, https://www.atcc.org) and the World Reference Center for Emerging Viruses and Arboviruses, University of Texas Medical Branch (https://www.utmb.edu/wrceva), to serve as the SARS-CoV-2 reference strain for the United States. The SARS-CoV-2 fourth passage virus has been sequenced and maintains a nucleotide sequence identical to that of the original clinical strain from the United States. These deposits make this virus strain available to the domestic and international public health, academic, and pharmaceutical sectors for basic research, diagnostic development, antiviral testing, and vaccine development. We hope broad access will expedite countermeasure development and testing and enable a better understanding of the transmissibility and pathogenesis of this novel emerging virus.
